# Selection of the best method for inherent tannin reduction and high nutrient retention

**DOI:** 10.4102/ojvr.v93i1.2236

**Published:** 2026-03-31

**Authors:** Sipho Silotolo, Nkosinathi F. Makhubela, Nhlamulo Chauke, Kedibone G. Mawela

**Affiliations:** 1 Department of Chemistry and Chemical Technology, School of Science and Technology, Sefako Makgatho Health Sciences University, Pretoria, South Africa; 2 Department of Biochemistry and Biotechnology, School of Science and Technology, Sefako Makgatho Health Sciences University, Pretoria, South Africa; 3 Department of Pharmacology and Therapeutics, School of Medicine, Sefako Makgatho Health Sciences University, Pretoria, South Africa

**Keywords:** antinutritive factors, legume seeds, nutrient retention, tannins, processing method

## Abstract

Raw legume seeds used in feed contain high antinutritive factors, such as tannins, which threaten livestock farming. Hence, the aim of the study was to (1) carry out single (raw plus dehulling [R + D]; soaking plus dehulling [S + D]; autoclaving plus dehulling [A + D]; infusion plus dehulling [I + D]; microwaving plus dehulling [M + D]; cooking plus dehulling [C + D] and combination [S + A + D; S + I + D; S + M + D; S + C + D; I + M + D, I + A + D]) processing methods on *Cicer arietinum, Arachis hypogaea*, and *Phaseolus vulgaris*; (2) determine dry weight yield and tannin reduction; (3) perform proximate and mineral analysis; (4) select the best method based on cost-effectiveness, high tannin reduction and high nutrient retention. The legume seeds were processed, dried, ground, and analysed for dry weight yield, tannin reduction, and proximate (ash, moisture, protein) and mineral (magnesium [Mg], zinc (Zn], copper [Cu], iron [Fe]) contents. A microplate reader and inductively coupled plasma were used as analytical tools. The results showed the highest (97.7%) and lowest (53%) dry weight yields in R + D and I + A + D, respectively. Effective tannin reductions were recorded in I + D (97.8%), S + D (93.9%), S + C + D (96.6%), S + M + D (92.2%), S + I + D (93.8), C + D (91.8%) and S + A + D (84.6%) methods with statistical significance (*p* < 0.05). Furthermore, ash, moisture, proteins and mineral retention had a range of 29% – 62% to indicate that tannin removal had no effect on nutrient compositions. Although S + M + D (62%) had the highest nutrient retention, S + D (53%) was selected as the best method as it satisfied all the requirements.

**Contribution:**

An effective processing method for use in rural farming to reduce tannins without compromising nutrition of the legume seeds was introduced.

## Introduction

In 2023, approximately 31.2% of people were living in rural settlements in South Africa (Food and Agriculture Organisation of the United Nations [FAO] n.d.), where livestock, especially ruminants, are the primary means of financial and food security. However, socio-economic and geographic factors play a significant role in the distribution of ruminant populations in these settlements. The resource-endowed farmers with sufficient land own large herds of cattle, sheep and goats for commercial purposes (Cooke et al. 2025). Their farming relies on commercial feed for livestock nutrition. Conversely, the low-resource smallholder farmers allow their livestock to graze in the rangeland where nutrition is not guaranteed, particularly during the feed scarcity period. Moreover, environmental conditions such as ultraviolet radiation, drought, flood and adverse temperatures often exacerbate nutrient-deficiency as they alter the chemical and nutrient (minerals, proteins and vitamins) compositions of forage (Rogowska & Szakiel 2020). Therefore, this form of farming results in low ruminant productivity, low standard of forage quality and quantity, diseases, parasites and limited access to veterinary therapeutics (Marandure et al. 2021). Because of its moderate livelihood sustainability, more efforts are in demand to improve livestock production systems.

Proteins play a central role in ruminant production systems, as it supports rumen microbial activity, tissue development, reproduction, immunity, and overall productivity. These are classified as rumen degradable protein (RDP) and rumen undegradable protein (RUP) (Putri et al. 2021). Enzymes secreted by ruminal bacteria, including but not limited to peptidases and proteases, degrade the RDP to turn it into peptides, amino acids and ammonia (NH_3_). Ammonia is converted into absorbable microbial crude protein, which is absorbed as small peptides and amino acids. While RUP is a true protein that is directly used by the ruminant without the need for rumen microbial degradation. As compared to microbial crude proteins, RUP provides high-quality amino acids, resulting in high-productive ruminants (Owens, Qi & Sapienza 2014).

Nevertheless, inadequate dietary protein results in reduced rumen microbial synthesis, leading to poor feed digestion, low weight gain, diminished milk yield, compromised fertility, and weakened immune responses (Besharati et al. 2022; Bešlo et al. 2022). It also increases the risk of metabolic disorders and delays growth in young ruminants, making protein-rich feed essential for both maintenance and production phases (Venegas-Calerón & Napier 2023).

Although protein requirements vary depending on physiological status, typically ranging from 10 to 16% crude protein in maintenance and growth diets, and higher levels during lactation or rapid growth phases (Department of Agriculture, Forestry & Fisheries [DAFF] n.d.), meeting these requirements is often challenging in rural communities where commercial protein supplements, such as soybean meal and fish meal, are costly or inaccessible (Snapp, Cox & Peter 2019). Hence, legume seeds present a practical and sustainable alternative. They are naturally rich in proteins and are well-adapted to African climatic conditions, including drought-prone and marginal environments (Morante-Carriel et al. 2024; Yang, Huang & Cao 2022). Crops such as chickpeas, peanuts, and beans can be cultivated locally with minimal inputs, reducing farmers’ reliance on purchased feed ingredients.

Despite the legume seeds’ nutritional potential, they contain antinutritive factors such as tannins that reduce protein digestibility and mineral availability in ruminants (Bocso & Butnariu 2022; Salam et al. 2023). Chronic exposure to high tannin levels can lead to gastrointestinal irritation, poor weight gain, and weakened immune function in livestock (Besharati et al. 2022; Bešlo et al. 2022). Addressing tannins in legume-based feed is vital for improving nutrient utilisation. Although traditional (soaking, cooking or boiling, steaming, roasting, infusion) and emerging (autoclaving, microwaving, fermentation, germination, enzyme treatment, pulse electric field, infrared cooking, extrusion cooking, ultrasound or sonication) processing methods have been used to reduce tannins (Badjona et al. 2023; Das, Sharma & Sarkar 2022 effective processing methods that reduce tannin levels without damaging nutrient content are critical for enabling smallholder farmers to use legumes as reliable protein sources in livestock feed.

Therefore, this study aimed to investigate and optimise the processing methods to find the best one that can be used to process the legume seeds as rich protein sources in animal feed and/or feed formulation. Such a method must satisfy the following requirements: (1) effective in tannin reduction; (2) retain high nutrients; (3) cost-effective. Thus, single processing methods (soaking, cooking, autoclaving, microwaving, and infusion) and their combinations were investigated. Proximate (ash, moisture and protein) and mineral (magnesium [Mg], zinc (Zn], copper [Cu], iron [Fe]) analysis were performed for quality and quantity purposes of the processed legume seeds.

## Research methods and design

### Sampling

For this study, only specific legume seeds were selected for analysis. The three legumes of interest that were selected include *Cicer arietinum* (chickpea) containing proteins, soluble and insoluble fibre, iron, folate, magnesium, zinc, vitamin B and antioxidants, *Arachis hypogaea* (chalimbana peanut) containing proteins, fats, fibre, vitamins, minerals and antioxidants, and *Phaseolus vulgaris* (red kidney bean), which contains proteins, fibre, iron, folate, magnesium, potassium and antioxidants. The selected legume seeds were purchased from Multisnack, South Africa. The ‘sample’ refers to the ground legume seeds (processed and unprocessed).

### Materials and apparatus

All analytical grade reagents and standards used in the study were purchased from Sigma-Aldrich (Johannesburg, South Africa) and Rochelle (Johannesburg, South Africa) while the microwave oven (Siemens, CM656GBS1, South Africa), weighing balance (Scientech, India), autoclave (Biobase Autoclave vertical steam steriliser, South Africa), FMH magnetic stirrer Str-MH, F7892-0318 with a hot plate, microplate reader SpectraMax ID3 (Molecular devices, United States), shaker (Labotec, South Africa), Kambrook Coffee & Spice Grinder (Clicks, South Africa), Whatman filter paper No 1, glassware and pipette were used.

### Processing of the legume seeds

All legume seeds (*C. arietinum, A. hypogaea*, and *P. vulgaris*) were processed immediately on arrival at the Sefako Makgatho Health Sciences University (SMU) Biochemistry laboratory. The initial processing included the removal of debris, damaged seeds and dust, rinsing, sun-drying, labelling, and storage at 4 °C to avoid microbial growth. Five single processing methods, namely, dehulling (D), cooking (C), soaking (S), autoclaving (A), microwaving (M), infusion (I) and their combinations were explored.

### Single processing methods

The processing methods were carried out as previously reported (Ayele et al. 2022; Chisowa 2022; Muhammed & Neme 2023) with slight modifications. All legume seeds were dehulled (R + D) by hand.

### Cooking plus dehulling (C + D)

All dehulled legume seeds were weighed at a ratio of 1:10 (weight/volume) into a glass beaker containing tap water and cooked on a hot plate at 100 °C for 30 min. This was done in triplicate. The cooked seeds were then dried at 80 °C for 12 h in a hot domestic oven, cooled to room temperature and ground into powder with a grinder.

### Autoclaving plus dehulling (A + D)

All dehulled *C. arietinum, A. hypogaea*, and *P. vulgaris* seeds were weighed at a ratio of 1:10 (weight/volume) into a glass beaker containing tap water and autoclaved at 1.5 × 10^6^ Pascal (Pa) at 121 °C for 20 min. This was done in triplicates. The autoclaved seeds were then dried at 80 °C for 12 h in a hot domestic oven, cooled to room temperature and ground into powder with a grinder.

### Microwave irradiation plus dehulling (M + D)

All dehulled legume seeds were weighed at a ratio of 1:10 (weight/volume) into a glass beaker containing tap water and microwave-heated above 600 W for 6 min using a domestic microwave. This was done in triplicate, and the microwaved seeds were dried at 80 °C for 12 h in a hot domestic oven, cooled to room temperature and ground into powder with a grinder.

### Infusion plus dehulling (I + D)

All dehulled legume seeds were weighed at a ratio of 1:10 (weight/volume) into a glass beaker containing tap warm water (60 °C). Then 2 g of sodium hydrogen carbonate (NaHCO_3_) and 1 g of citric acid (C_6_H_8_O_7_) were added. The mixture was infused for 30 min. This was done in triplicate. The infused legume seeds were then dried at 80 °C in a hot domestic oven for 12 h, cooled to room temperature and ground into powder with a grinder.

### Soaking plus dehulling (S + D)

All dehulled legume seeds were weighed at a ratio of 1:10 (weight/volume [w/v]) into a glass beaker containing distilled water and soaked for 12 h. The soaked legume seeds were then dried at 80 °C in a hot domestic oven for 12 h, cooled to room temperature and ground into powder with a grinder.

### Combined processing methods

Six combined processing methods, namely, S + C + D, S + A + D, S + M + D, S + I + D, I + M + D and I + A + D were investigated. These were performed by combining the single processing methods. The first two letters in a combined method provide the order or sequence of the dehulled legume seed processing.

### Tannin extraction and analysis

The method was carried out as previously reported with slight modifications (Asante et al. 2024). All processed and raw legume seed powders were separately weighed at a ratio of 1:10 (weight/volume) in ethanol and extracted on a shaker set at 150 rate per minute (rpm) for 24 h with regular monitoring every 3 h during the day and continued with the same speed throughout the night. The mixture was allowed to settle down and filtered with Whatman No. 1 filter paper. The process was repeated three times.

The supernatant was collected and used for the determination of tannin contents using the Folin–Ciocalteu method. About 100 µL of the supernatant was added to a 10 mL volumetric flask containing 7.5 mL of distilled water, 0.5 mL of Folin–Ciocalteu reagent and 20% of sodium carbonate. The mixture was diluted to 10 mL with distilled water, shaken well and kept at room temperature for 30 min. A set of reference standard solutions of tannic acid (10 µg/mL – 100 µg/mL) was prepared, and the absorbance for test and standard solutions was read against the blank at 550 nanometre using a microplate reader. Tannin concentrations were determined by interpolation from the drawn calibration curve. To calculate tannin reduction, the tannin concentration of the raw seeds was compared with that in all the processed legume seeds and presented as percentages using the following formula (Equation 1):


$$\%\text{Tannin reduction}=\left(\frac{\text{Craw}-\text{Cprocessed}}{\text{Craw}}\right)\times 100$$ [Eqn 1]

### Determination of nutritional value of the processed legume seeds

Apart from proteins, proximate analyses (ash and moisture) were performed following the Official Methods of Analysis of AOAC Official Methods (925.10, 923.03 2023). While the mineral digestion procedures were performed according to the United States Environmental Protection Agency (EPA) method (1996).

#### Ash content

The method was carried out as previously reported with slight modifications (AOAC Official Method 923.03 2023). A crucible and lid were weighed, and 1 g of a sample was added. The crucible was heated for 2 h in a muffle furnace at 550 °C to burn off organic material, leaving ash. The hot crucible was removed from the furnace, cooled in a desiccator and weighed. The ash content was calculated using the following formula (Equation 2):


$$\%\text{Ash content}=\left(\frac{\text{weight of ash}\left[\text{g}\right]}{\text{weight of sample}\left[\text{g}\right]}\right)\times 100$$ [Eqn 2]

#### Moisture content

In order to guarantee good taste, texture and shelf-life, the dry processed legume seeds should retain quality, chemical stability and safety from microbial infection. This method was carried out as previously reported with slight modifications (AOAC Official Method 925.10 2023). An empty, dry glass beaker was pre-weighed, and the weight was recorded. An exact 1 g of the dry processed legume seeds from each processing method was weighed on a pre-weighed glass beaker, then dried in an oven at 105 °C for 2 h. The hot dish was removed from the oven, cooled in a desiccator and then dried. Moisture content was calculated using the following formula (Equation 3):


$$\%\text{Moisture content}=\left(\frac{\begin{array}{l} \text{Initial weight}\left[\text{g}\right]-\\ \text{Weight after drying}\left[\text{g}\right] \end{array}}{\text{Initial weight}\left[\text{g}\right]}\right)\times 100$$ [Eqn 3]

#### Mineral analysis on inductively coupled plasma optical emission spectroscopy

The method was carried out according to the previous report, which was slightly modified (EPA Test Method 3052 1996; Zasoski & Burau 1977). Half a gram of the sample was digested with 7 mL of concentrated nitric acid (HNO_3_) and 3 mL of perchloric acid (HClO_4_) at a temperature of 180 °C. The digestion was brought to volume in a 100 mL volumetric flask. An aliquot of the digest was subjected to inductively coupled plasma optical emission spectroscopy (ICP-OES) analysis with an Agilent 725 simultaneous instrument, where wavelengths of four elements (Mg, Fe, Zn, Cu) were determined simultaneously. Each element was measured at one or two appropriate emission wavelengths, chosen for high sensitivity and lack of spectral interferences. The instrument was set up and operated as per the manufacturer’s specifications. It was calibrated against a series of standard solutions containing the four elements of interest following optimised procedures based on the instrument manual and in-house validated and unpublished methods of the Agricultural Research Council - Institute for Soil, Climate and Water (ARC-ISCW). The operating parameters are presented in Table 1. The results were represented as mineral concentrations (milligram per kilogram [mg/kg] or percentage), while relative standard deviation (RSD) was used to measure the precision of the mineral replicates.

**TABLE 1:** Inductively coupled plasma optical emission spectroscopy operation parameters.

Parameter	Settings
Radio frequency power (kW)	1.00
Plasma flow (L/min)	15.00
Auxiliary flow (L/min)	1.50
Nebuliser flow (L/min)	0.55
Pump rate (rpm)	15.00
Viewing Height (mm)	14.00
Instrument delay (s)	15.00
Sensitivity	Wide range
Sample uptake delay (s)	35.00
Fast pump (sample delay/rinse)	√
Standards	Zn; Fe; Mg; Cu
Rinse time (s)	25.00
Replicate read time (s)	4.00
Replicates	3.00

Mg, magnesium; Zn, zinc; Cu, copper; Fe, iron.

#### Protein analysis

The Bradford assay was used to estimate total protein content as previously reported with slight modifications (Dent, LeMinh & Maleky 2024; Giangrieco et al. 2022). Briefly, 1 g of the legume sample was weighed into a glass beaker and about 15 mL of 80% aqueous methanol was added. The mixture was placed on a shaker set at 150 rate per minute (rpm) for 24 h with regular monitoring every 3 h during the day and continued at the same speed throughout the night. The mixture was allowed to settle down and filtered with Whatman No. 1 filter paper and the supernatant was collected. The process was repeated three times. Protein solutions were made at 1% w/v by diluting the supernatant 10× in deionised water. Then, 100 µL of the diluted supernatant was loaded onto 96-well plate in triplicate. Then, 100 µL of Bradford reagent was added. Bovine saline albumin solutions (30 mg/mL – 240 mg/mL) were prepared as standards and treated the same as the extracts. The plate was incubated for 5 min, then absorbance was measured at 490 nm using a microplate reader. A calibration curve was drawn with standard concentrations for interpolation purposes, and the results were expressed as g/100g.

### Data analysis

All analysis was conducted in triplicate, and the results were expressed as means and standard deviations (s.d.). Analysis of variance (ANOVA) was used to determine whether there is a significant difference among the means of the processed legume seeds. The processed legume seeds were then compared against each other relative to the raw using the Tukey post hoc test at 95% confidence. Processed methods that are not significantly different at the chosen alpha level share a letter.

For proximate and mineral compositions, the Mann–Whitney *U* test and one-way ANOVA were used to compare 2 groups and ≥ 3 groups, respectively. Only processed legume seeds with high nutrient content were subjected to analysis.

### Ethical considerations

No animal was used in the study. Ethical clearance to conduct this study was obtained from the Sefako Makgatho University Research Ethics Committee (No. REC 210408-003, SMUREC/ S/472/2024:PG).

## Results

### Effects of the processing methods on dry weight yield and tannin reduction

**FIGURE 1:**
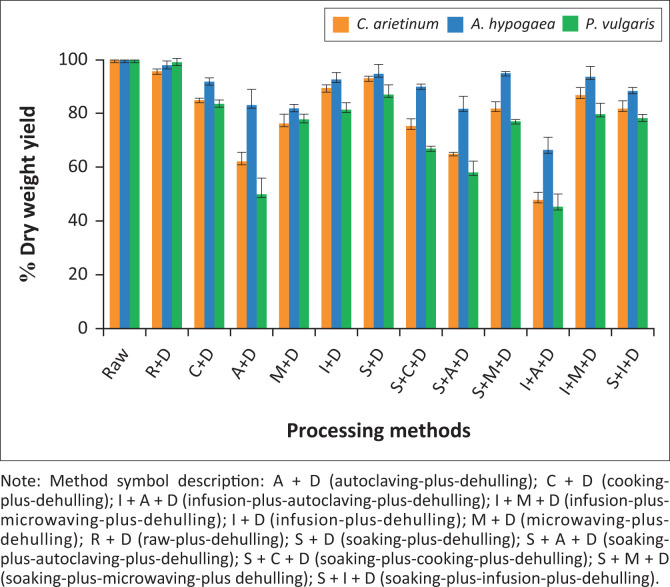
Percentage dry weight yields of the processing methods across different legume seeds.

The percentage dry weight yield of *C. arietinum, A. hypogaea*, and *P. vulgaris* subjected to different processing methods was determined as illustrated in Figure 1. The R + D treatment of all processed legume seeds, with an average of 97.7%, resulted in the highest percentage yields of 95.7%, 98.2% and 99.2% in *C. arietinum, A. hypogaea* and *P. vulgaris*, respectively. In addition, the highest yields of 94.8%, 95.1% and 95.1% were shown in S + D, S + M + D and I + M + D of *A. hypogaea*, respectively. While S + D in *C. arietinum* showed the highest yield of 93.2%. Contrariwise, the lowest percentage yields of 50% (A + D), 48.01% (I + A + D) and 45.1% (I + A + D) that averaged to 53%, were obtained in *P. vulgaris, C. arietinum* and *P. vulgaris*, respectively.

On the other hand, tannin reduction of the processing methods was determined as illustrated in Table 2. Thus, various calibration plots that show regression equations with correlation coefficients were drawn, and the results were not shown. However, the plots showed good linearity over the range tested, and the correlation coefficients were around 0.975. Nevertheless, three categories of tannin reductions were considered, namely, ineffective (0% – 50.9%); moderate (51% – 79.9%); effective (80% – 100%). Therefore, *C. arietinum* (R + D: 17.6%; A + D: 43.88%; M + D: 24.11%; I + A + D: 46.03%) and *P. vulgaris* (M + D: 39.54%; R + D: 30.41%) and *A. hypogaea* (R + D: 14.55) were ineffective. While *C. arietinum* (C + D: 78.18%; S + A + D: 63.65%) and *A. hypogaea* (I + A + D: 53.84%; A + D: 60.82%; I + M + D: 61.69%; M + D: 70.46%) showed moderate tannin reduction. The rest of the methods in *C. arietinum* (I + D; S + D; all soakings except S + A + D; I + M + D), *A. hypogaea* (C + D; I + D; all soakings) and *P. vulgaris* (C + D; all infusions and soakings) showed effective tannin reduction, with percentage values exceeding 80%. Therefore, 50% of the single processing methods and 67% of the combined methods explored in the study were effective in reducing the tannins. Hence, they were further analysed for proximate and mineral content.

**TABLE 2:** Tukey’s post hoc test results for comparing percentage tannin reductions of the processed seeds.

Legume seed	Processing method	*N*	Mean	s.d.	s.e.	Coefficient of variation	Tukey grouping
*Arachis hypogaea*	Raw	3	0.00	0.00	0.00	NaN	a
	A + D	3	60.73	0.73	0.42	0.01	h
	C + D	3	97.40	1.31	0.76	0.01	nop
	I + A + D	3	53.55	0.29	0.17	0.02	g
	I + D	3	97.21	1.75	1.01	0.00	nop
	I + M + D	3	61.70	0.29	0.17	0.33	h
	M + D	3	17.46	5.82	3.36	0.33	bc
	R + D	3	13.58	4.45	2.57	0.00	bc
	S + A + D	3	91.77	0.34	0.19	0.00	klmn
	S + C + D	3	94.68	0.17	0.10	0.01	nop
	S + D	3	88.28	0.44	0.26	0.00	jkl
	S + I + D	3	94.20	0.44	0.26	0.01	lmnop
	S + M + D	3	92.06	1.02	0.59	0.01	klmno
*Cicer arietinum*	Raw	3	0.00	0.00	0.00	NaN	a
	A + D	3	44.46	0.61	0.35	0.01	f
	C + D	3	78.41	0.61	0.35	0.01	i
	I + A + D	3	46.50	0.47	0.27	0.01	f
	I + D	3	96.45	1.83	1.06	0.02	nop
	I + M + D	3	88.60	0.64	0.37	0.01	jklm
	M + D	3	24.89	0.92	0.53	0.04	d
	R + D	3	18.06	4.60	2.66	0.25	c
	S + A + D	3	63.93	1.07	0.62	0.02	h
	S + C + D	3	97.57	0.18	0.10	0.00	nop
	S + D	3	94.31	0.92	0.53	0.01	mnop
	S + I + D	3	92.27	1.38	0.80	0.01	klmno
	S + M + D	3	87.18	1.68	0.97	0.02	jk
*Phaseolus vulgaris*	Raw	3	0.00	0.00	0.00	NaN	a
	A + D	3	87.09	0.34	0.20	0.00	jk
	C + D	3	99.69	0.29	0.16	0.00	p
	I + A + D	3	96.77	0.14	0.08	0.00	nop
	I + D	3	99.74	0.21	0.12	0.00	p
	I + M + D	3	85.17	0.69	0.40	0.01	j
	M + D	3	37.86	4.94	2.85	0.13	e
	R + D	3	30.24	2.06	1.19	0.07	d
	S + A + D	3	11.84	1.37	0.79	0.12	b
	S + C + D	3	97.36	0.21	0.12	0.00	nop
	S + D	3	99.46	0.21	0.12	0.00	p
	S + I + D	3	97.86	0.27	0.16	0.00	op
	S + M + D	3	97.22	0.96	0.56	0.01	nop

Note: Different letters indicate statistical significance (*p* < 0.05). Method symbol description: A + D (autoclaving-plus-dehulling); C + D (cooking-plus-dehulling); I + A + D (infusion-plus-autoclaving-plus-dehulling); I + D (infusion-plus-dehulling); I + M + D (infusion-plus-microwaving-plus-dehulling); M + D (microwaving-plus-dehulling); R + D (raw-plus-dehulling); S + A + D (soaking-plus-autoclaving-plus-dehulling); S + C + D (soaking-plus-cooking-plus-dehulling); S + D (soaking-plus-dehulling); S + I + D (soaking-plus-infusion-plus-dehulling); S + M + D (soaking-plus-microwaving-plus dehulling).

NaN, not a number; s.d., standard deviation; s.e., standard error.

### Proximate analysis

As part of the proximate analysis, the ash, moisture and protein contents were determined (Table 3). The proximate data for ash and moisture were expressed in percentage (%). Apart from S + C + D (*A. hypogaea*), all ash contents were between 1% and 4%. Similarly, the % moisture contents ranged between 2.5% and 3.5%, except for C + D, I + D and S + C + D, which were less than 2% but greater than 1%.

**TABLE 3:** Proximate compositions with standard deviations of the legume seeds subjected to different processing methods.

Processing method	Ash (%)			Moisture (%)			Proteins (g/100g)		
	CA	AH	PV	CA	AH	PV	CA	AH	PV
Raw	3.01 ± 0.03	4.01 ± 0.01	4.00 ± 0.02	3.50 ± 0.01	2.82 ± 0.12	2.50 ± 0.03	137 ± 1.12	279 ± 0.69	419 ± 4.15
C + D	2.22 ± 0.02	1.11 ± 0.01	1.09 ± 0.02	2.74 ± 0.01	3.07 ± 0.11†	1.58 ± 0.00	148 ± 3.65*†	275 ± 1.01	362 ± 2.30
I + D	1.01 ± 0.01	3.23 ± 0.02	4.07 ± 0.02*†	2.75 ± 0.01	1.20 ± 0.02	2.71 ± 0.02†	153 ± 2.08*†	354 ± 3.00*†	315 ± 0.55
S + D	2.14 ± 0.01	2.20 ± 0.01	3.14 ± 0.01	2.85 ± 0.10	3.05 ± 0.31†	3.57 ± 0.12†	158 ± 2.88*†	310 ± 2.97*†	430 ± 2.60*†
S + A + D	1.03 ± 0.06	2.08 ± 0.01	1.23 ± 0.02	2.94 ± 0.03	3.64 ± 0.36†	2.86 ± 0.04†	108 ± 1.81*†	257 ± 0.99*	309 ± 0.84
S + C + D	1.00 ± 0.01	8.09 ± 2.00	2.05 ± 0.02	2.52 ± 0.09	2.68 ± 0.32	1.05 ± 0.00	146 ± 2.76*†	310 ± 6.90*†	393 ± 0.43
S + M + D	2.00 ± 0.02	1.13 ± 0.01	4.03 ± 0.03*†	2.77 ± 0.03	3.19 ± 0.29†	2.82 ± 0.06†	149 ± 0.77*†	343 ± 1.31*†	454 ± 0.72*†
S + I + D	2.06 ± 0.01	3.07 ± 0.02	4.11 ± 0.02*†	3.53 ± 0.11†	2.75 ± 0.28	2.45 ± 0.21	164 ± 5.93*†	337 ± 3.00*†	433 ± 0.08*†

Note: Method symbol description: C + D (cooking-plus-dehulling); I + D (infusion-plus-dehulling); S + D (soaking-plus-dehulling); S + A + D (soaking-plus-autoclaving-plus-dehulling); S + C + D (soaking plus-cooking-plus-dehulling); S + M + D (soaking-plus-microwaving-plus-dehulling); S + I + D (soaking-plus-infusion-plus-dehulling).

AH, *Arachis hypogaea*; CA, *Cicer arietinum*; PV, *Phaseolus vulgaris*; ANOVA, analysis of variance.

* , Indicates statistical significance (*p* < 0.05), where the Mann–Whitney *U* test and one-way ANOVA were used for comparing 2 groups and ≥ 3 groups, respectively.

† , Indicates high nutrient relative to raw.

Conversely, the total proteins were expressed in g/100g. The standard curve that shows regression equation and correlation coefficient was drawn and used to extrapolate protein contents of the processed legume seeds. Although the calibration plot is not shown, a high regression coefficient of 0.9907 indicates a good relationship between the absorbance and the tested concentration range. Nonetheless, *P. vulgaris* showed the highest protein contents across the processing methods except in I + D. This was followed by *A. hypogaea*, while *C. arietinum* had the lowest protein content across the processing methods.

### Inductively coupled plasma optical emission spectroscopy mineral analysis

Four mineral (Fe, Mg, Cu and Zn) contents were determined. The Zn, Fe and Cu contents were expressed in mg/kg, while Mg was expressed as a percentage (Table 4). *Cicer arietinum* showed higher Zn contents in the processed legume seeds than in the raw seed (32.5 mg/kg). Some processed *A. hypogaea* and *P. vulgaris* legume seeds contained higher Zn contents while others had lower Zn contents than their raw seeds (41.7 mg/kg [*A. hypogaea*]; 29.9 mg/kg [*P. vulgaris*]). Nevertheless, the highest Zn contents of 51.4 mg/kg (I + D) and 34.5 mg/kg (S + C + D) were determined in *A. hypogaea* and *P. vulgaris*, respectively.

**TABLE 4:** Mineral content in the legume seeds with relative standard deviation < 10%.

Processing method	Mg (%)			Zn (mg/kg)			Cu (mg/kg)			Fe (mg/kg)		
	CA	AH	PV	CA	AH	PV	CA	AH	PV	CA	AH	PV
Raw	0.14	0.14	0.14	32.5	41.7	29.9	7.41	5.79	11.40	82.9	27.0‡	70.9
C + D	0.11	0.22†	0.10	48.6†	34.3	27.4	7.62†	8.13†	9.50	101.0†	28.9†	70.5†
I + D	0.09	0.22†	0.11	42.2†	51.4†	26.6	6.88	8.24†	8.87	59.0	48.3†	74.7†
S + D	0.14†	0.20†	0.10	34.1†	33.5	25.9	8.12†	7.34†	8.21	74.8‡	32.5†	81.8†
S + A + D	0.09	0.21†	0.08	35.1†	41.8†	26.6	6.91	5.93†	8.99	79.0	28.5†	65.3
S + C + D	0.09	0.20†	0.09	37.2†	32.3	34.5†	6.76	6.06†	8.23	62.4	38.8†	80.4†
S + M + D	0.11	0.17†	0.11	43.7†	30.9	28.9	6.50	6.40†	7.83	84.7†	55.3†	86.2†
S + I + D	0.10	0.20†	0.10	33.3†	35.2	25.7	7.22	7.79†	8.69	62.7	26.0	75.8†

Note: Method symbol description: C + D (cooking-plus-dehulling); I + D (infusion-plus-dehulling); S + D (soaking-plus-dehulling); S + A + D (soaking-plus-autoclaving-plus-dehulling); S + C + D (soaking-plus-cooking-plus-dehulling); S + M + D (soaking-plus-microwaving-plus-dehulling); S + I + D (soaking-plus-infusion-plus-dehulling). Results were not statistically significant (*p* > 0.05).

AH, *Arachis hypogaea*; CA, *Cicer arietinum*; Cu, copper; Fe, iron; Mg, magnesium; PV, *Phaseolus vulgaris*; Zn, zinc; RSD, relative standard deviation.

† , High mineral contents relative to the raw seeds;

‡ , Indicates RSD > 10 %.

All Cu contents in the processed *P. vulgaris* were relatively low as compared to the raw seed that contained 11.4 mg/kg of Cu. However, the highest Cu content of 7.83 mg/kg was determined in S + M + D. Furthermore, the Fe content of *C. arietinum* raw seed (82.9 mg/kg) was lower than that of C + D (101 mg/kg) and S + M + D (84.7 mg/kg). Although *A. hypogaea* raw seeds had a low Fe content of 27 mg/kg, it was slightly higher than the S + I + D (26 mg/kg) Fe content. It is notable that the highest Fe content (55.3 mg/kg) was obtained in S + M + D. For *P. vulgaris*, the Fe content of raw seeds was 70.9 mg/kg. This was slightly higher than the C + D (70.5 mg/kg) and S + A + D (65.3 mg/kg) Fe content. Otherwise, the highest Fe content of 86.2 mg/kg was obtained in S + M + D.

All processed *A. hypogaea* seeds had higher Mg contents that ranged between 0.174% and 0.218% than the raw seeds (0.143%). Mg content of 0.136% of *C. arietinum* raw seeds was found to be higher than that of the processed legume seeds, which ranged from 0.091 to 0.112%. On the other hand, *P. vulgaris* raw seeds had a higher Mg content of 0.141% than all the processed legume seeds. The highest Mg content of 0.111% was determined in S + M + D, while 0.081% was the lowest content in S + A + D.

### Selection of the best method

Furthermore, the experimental trials (proximate composition) of all the processed legume seeds were used to determine the percentage of high nutrient retention (Table 5). Henceforth, the methods were classified under three categories based on their nutrient retention relative to the respective raw seeds, namely, good (0% – 29.9%); better (30% – 59.9%) and best (60% – 100%). It is worth noting that the processing methods with the same nutrient retention as the raw seeds were considered as high retention. From the results obtained, S + A + D showed good nutrient retention. The methods with better nutrient retention were S + D, I + D, C + D, S + C + D and S + I + D, while S + M + D showed the best nutrient retention.

**TABLE 5:** Nutrient retention of legume seeds relative to the respective raw seeds.

Processing method	High retention trials		Mg (%)			Zn (mg/kg)			Cu (mg/kg)			Fe (mg/kg)			Ash (%)			Moisture (%)			Protein (g/100g)		
	*n*	%	PV	CA	AH	PV	CA	AH	PV	CA	AH	PV	CA	AH	PV	CA	H	PV	CA	AH	PV	CA	AH
I + D	9	43	L	L	L	L	H	H	L	L	H	H	L	H	SR	L	L	H	L	L	L	H	H
S + D	11	53	L	H	L	L	H	L	L	H	H	H	L	H	L	L	L	H	L	H	H	H	H
C + D	9	43	L	L	H	L	H	L	L	H	H	L	H	H	L	L	L	L	H	H	L	H	L
S + A + D	6	29	L	L	H	L	H	H	L	L	H	L	L	H	L	L	L	L	L	H	L	L	L
S + C + D	11	53	L	L	H	H	H	H	L	L	H	H	L	H	L	L	H	L	H	L	H	L	H
S + M + D	13	62	L	L	H	L	H	L	L	L	H	H	H	H	SR	L	L	H	H	H	H	H	H
S + I + D	9	43	L	L	H	L	H	L	L	L	H	H	L	L	SR	L	L	L	H	L	H	H	H

Note: Method symbol description: C + D (cooking-plus-dehulling); I + D (infusion-plus-dehulling); S + D (soaking-plus-dehulling); S + A + D (soaking-plus-autoclaving-plus-dehulling); S + C + D (soaking-plus-cooking-plus-dehulling); S + M + D (soaking-plus-microwaving-plus-dehulling); S + I + D (soaking-plus-infusion-plus-dehulling).

AH, *Arachis hypogaea*; CA, *Cicer arietinum*; Cu, copper; Fe, iron; H, high nutrient retention; L, low nutrient retention; Mg, magnesium; PV, *Phaseolus vulgaris*; SR, same nutrient retention with raw seed; Zn, zinc.

## Discussion

### Processing effects on dry weight yield and tannin reduction

Percentage dry weight yield varied across legumes, with R + D and S + D showing the highest yield (Figure 1). In contrast, methods such as A + D and I + A + D led to lower yields in *P. vulgaris* and *C. arietinum*, likely because of loss of seed components and leaching of nutrients. *Arachis hypogaea* showed minimal mass loss, likely because of its high fat content and dense structure, which reduces nutrient leaching and breakdown (Gelaye & Luo 2024). Whereas *C. arietinum* and *P. vulgaris*, which are richer in proteins and carbohydrates, were more prone to leaching and degradation, especially under intense treatments such as I + A + D because soluble carbohydrates, such as oligosaccharides, can be easily lost in soaking or cooking water, thereby reducing final dry weight. In addition, proteins may denature and diffuse out of the seed structure when exposed to high temperatures and pressure, especially when seed coats are compromised (Alarape et al. 2024).

These variations highlight the impact of structural and compositional factors such as protein, carbohydrate, and fat content on yield stability (Alarape et al. 2024; Etzbach et al. 2024). Therefore, optimising the conditions for the processing methods to minimise mass loss while preserving nutritional properties is crucial, and further research should investigate the biochemical mechanisms affecting yield retention.

With respect to tannin reduction (Table 2), the results showed that different processing methods reduced tannin contents differently, and some reductions were statistically significant (*p* < 0.05). Hence, three tannin reduction categories, namely, ineffective (0% – 50%); moderate (51% – 79%) and effective (80% – 100%), were implemented in the study.

Under the ineffective tannin reduction category, R + D was found to be ineffective across all three legume seeds, indicating that the dehulling process on its own is insufficient in reducing the tannins. Although tannin reductions in *C. arietinum* and *A. hypogaea* were lower than in *P. vulgaris*, they were statistically significant (*p* < 0.05). Furthermore, tannin distribution in the three main parts of the seeds namely, the seed coat (hull), germ and cotyledon, varies across different legume seeds (Ajala et al. 2023).

As tannins exist as hydrolysable and condensed (proanthocyanidins) forms, the former tannins are easily hydrolysable as they consist of hydrolysable ester bonds, while the latter are not hydrolysable because of the presence of C-C or C-O-C bonds (Bule et al. 2020). Thus, hydrolysable tannins can leach out easily, whereas condensed tannins may require traditional processing methods such as microwaving, cooking or autoclaving to boost their solubility to leach out. Moreover, as tannins are polyphenolic in nature, they leach out into the soaking water under the influence of a concentration gradient (Malenčić, Cvejić & Miladinović 2012). Henceforth, one traditional processing method may not be effective in reducing the tannins on all legume seeds due to variations and the nature of these tannins found in these legume seeds. For instance, the A + D (*C. arietinum*), M + D (*C. arietinum* and *P. vulgaris*) and I + A + D (*C. arietinum*) processing methods were ineffective for the legume seeds indicated in brackets, and the tannin reductions had no statistical significance (*p* > 0.05).

On the other hand, other processing methods like C + D (*C. arietinum*), A + D (*A. hypogaea*), M + D (*A. hypogaea*), S + A + D (*C. arietinum*) and I + A + D (*C. arietinum* and *A. hypogaea)* showed moderate tannin reduction where some results had statistical significance (*p* < 0.05). In contrast, the results of other methods in *C. arietinum* (I + D; S + D; S + C + D; S + M + D; S + I + D; I + M + D), *A. hypogaea* (C + D; I + D; S + D; S + C + D; S + A + D; S + M + D; S + I + D) and *P. vulgaris* (C + D; S + M + D; S + I + D; I + A + D; I + D; S + D; S + C + D; S + A + D), which showed effective reduction, were in line with the previous studies. For instance, Helbig et al. (2003b) investigated the effect of soaking prior to cooking on tannin levels of *P. vulgaris* and found that cooking without prior soaking reduced tannins by 86.6%, while cooking with the non-absorbed soaking water reduced tannins by 89.0%. Furthermore, the microwaving and autoclaving methods had less impact on tannin reduction in *C. arietinum*.

With regard to the processing of *A. hypogaea* seeds, other studies that used the boiling method exhibited the lowest tannin content reduction of 50.85% and 44.63% in Sodari and Madani peanuts, respectively (Eltom, Sulieman & Hassan 2023), which is lower than 91.8% obtained in this study. These findings suggest that effective hydration and thermal methods are key for tannin reduction in peanuts. Another study that showed low tannin reduction range of 5.27% – 3.33% (approximately 36.79%) was reported when the soaking method was used on *A. hypogaea* seeds (Etti & Adie 2022). Contrariwise, the infusion-based methods (I + D) reduced tannins by 95%, emphasising the role of solvent-assisted extraction, as previously reported in a study on black grams (*Vigna mungo*), where soaking in sodium bicarbonate solution led to a tannin reduction of approximately 55% (Rehman & Shah 2001). In addition, Vadivel and Biesalski (2012) in a separate study reported that soaking in alkaline solutions followed by cooking resulted in tannin reductions ranging between 33% and 51%, with some legumes showing reductions of up to 78%. Otherwise, the microwave method combined with other techniques, such as the infusion and dehulling (I + M + D), had a moderate effect (55%) on tannin reduction. This is because tannins are heat-sensitive, and heating processing methods such as microwaves often cause substantial reductions (Sharma et al. 2018). Thus, the microwave process can reduce tannins as it breaks down the tannin–protein complex and degrades the heat-sensitive free tannins (Das et al. 2025).

In terms of *P. vulgaris*, the processing methods resulted in consistently higher tannin reductions. The I + D reduced tannins by a remarkable 99.78%. This was followed closely by S + D at 99.51%, highlighting the effectiveness of both soaking and infusion in this study. But then, 46.88% and 54.76% tannin reduction in *P. vulgaris* were previously reported in soaking and soaking with sodium bicarbonate, respectively (Nagessa et al. 2023). Another study that explored the effect of processing in three kidney bean species showed that S + D reduced tannins by 23% – 30% while cooking reduced tannins by 21% – 41%, emphasising that hydration alone is highly effective in the common beans (Abera, Yohannes & Chandravanshi 2023). Moreover, the investigation of the effect of soaking and cooking of *P. vulgaris* previously reported a 65.81% tannin reduction in the sodium bicarbonate soaking plus cooking method (Huma et al. 2008).

In general, the M + D (27%) method was found to be ineffective in tannin reduction of the three legume seeds investigated. The I + M + D (78.8%), I + A + D (65.6%) and A + D (63.9%) showed moderate reduction. Apart from the infusions that showed moderate reduction, all hydration-based methods namely, I + D (97.8%), S + D (93.9%), S + C + D (96.6%), S + M + D (92.2%), S + I + D (93.8), C + D (91.8%) and S + A + D (84.6%) were effective in reducing the tannins across all legume seeds. These results show that most tannins in these legume seeds could be hydrolysable in nature, and the traditional processing methods in combination with the microwave, autoclave and infusion were effective in increasing solubility in the case of the condensed tannins. Because the autoclave, as a single method, was the least effective in this study, autoclave time can be optimised to obtain better results in the same way as it was done previously. A typical example was a study that investigated the effective autoclaving time required to reduce the tannins in three legume grains namely, cowpea, pigeon pea and soybean (Chisowa 2022). The results showed that autoclaving for 3 min reduced the tannin contents in cowpea, pigeon pea and soybean by 37.5%, 54.2% and 46.7%, respectively. However, when autoclaving time was extended to 6 min, tannin reduction further increased to 65%, 65.1% and 71.1% in cowpea, pigeon pea and soybean, respectively.

### Proximate composition

#### Ash content

Analysis of ash content revealed significant trends regarding mineral retention across various processing methods. The *C. arietinum* raw seeds had an ash content of 3.2%. All ash contents of the processed legume seeds ranged between 1% and 2%. The low ash contents may be because of mineral leaching into the cooking water of C + D. In the case of S + D, the low 2% ash content may be attributed to mineral leaching during the soaking process, as water-soluble minerals diffuse into the soaking medium and are subsequently lost when water is discarded. Contrariwise, previous studies reported a slightly higher ash contents in the chickpeas that indicate better mineral retention capacity. For instance, Mathew, Sumi and Shakappa (2024) reported ash contents of 1.88% – 3.11% from 33 varieties of chickpeas that were analysed, while Imwinkelried et al. (2025) reported 4.3% content. On the other hand, another study investigated the effect of cooking and different storage conditions on the crude ash of the chickpea (Chauhan et al. 2025). The results showed that the freshly prepared chickpeas contained 2.33% crude ash, while the chickpeas stored at room temperature for 24 h and those that were kept at 4 °C for 24 h contained 2.63% and 2.67%, respectively.

In *A. hypogaea*, the raw ash content of 4%, while S + M + D and S + I + D showed reductions of 1% and 3%, respectively. A notable increase to 8% (confirmed through repeated analysis) was observed in S + C + D, suggesting that this method may significantly elevate ash content in some legumes. Conversely, a previous study that carried out an investigation of the effects of processing on the proximate composition of *A. hypogaea* reported the ash contents of 2.4%, 3.25% and 2.82% in raw, boiled and fried groundnuts, respectively (Sanni et al. 2024). Although roasting was not explored in this study, El Idrissi et al (2024) reported an increase in ash content from mean 1.28 s.d. ± 0.01% to mean 5.45 s.d. ± 0.05% after the influence of thermal processing and varietal disparities on the nutritional composition of peanut flour and oilcake flour was investigated. These results show that roasting has the potential to retain mineral contents in *A. hypogaea*.

For *P. vulgaris*, raw seeds had an ash content of 4% while methods such as S + A + D reduced ash to 1%, suggesting mineral leaching during soaking and autoclaving. Because of high mineral loss, legume seeds processed with methods such as S + A + D may be unsuitable for use as feed formulations for ruminants and non-ruminants. Otherwise, I + D, S + I + D, and S + M + D retained higher ash contents (4%). Nonetheless, Roy et al. (2021) reported higher ash contents of mean 4.82 s.d. ± 0.27% (raw) and mean 3.35 s.d. ± 0.42% (boiled) in dark red kidney beans. Thus, variation of the raw ash contents could be because of harvest season, post-harvest processing before entering the market or different geographic locations where these seeds were grown. The *P. vulgaris* seeds from Roy et al. (2021) were purchased from the local market of Sylhet, Bangladesh, South Asia. In this study, they were purchased from Multisnack in Johannesburg, South Africa. With respect to the single methods, previous studies investigated the impact of dehulling and soaking of *P. vulgaris* that led to ash content decrease from 4.1% (raw seeds) to 3.6% (dehulled or soaked) (Viana & Marcia 2022)(Viana Lauren & Marcia, 2022). On the other hand, Helbig, Da Costa Peres and De Carvalho (2003a) observed ash contents ranging from 3.5% to 4.1% in common beans cooked in their soaking water. In contrast, Mubarak (2005) found a decrease in ash content from 3.0% to 2.4% in lentils following soaking and cooking in water, attributing the mineral loss to leaching.

In addition, other types of beans where the effect of traditional processing methods on *V. radiata L* was investigated, the boiled seeds had a lower ash content (mean 2.32 s.d. ± 0.02) compared to raw (mean 4.76 s.d. ± 0.02%) and dehulled (mean 4.31 s.d. ± 0.04%) samples (Tura et al. 2023). This suggests that the seed coat has a lot of minerals; hence, dehulling resulted in a decrease in ash content in lima beans. Another study that investigated the effect of different processing methods on the proximate content of lima beans reported that soaking for 24 h led to an 18.9% increase in ash content, while cooking unsoaked seeds increased the ash content to 3.8% (Jayalaxmi et al. 2015). Autoclaving soaked seeds increased ash content by 11.3%, while dehulling caused a 9.4% rise. These results indicate that some processing methods can boost ash content in lima beans and other legume seeds explored in this study.

#### Moisture content

Moisture content affects shelf life, microbial stability, and processing of legume seeds. In this study, *C. arietinum, A. hypogaea*, and *P. vulgaris* showed moisture contents between 1.05% and 3.64%. As raw seeds were purchased from the market, it is presumed that they have gone through rigorous post-harvest processing, monitoring, validations, optimisations for safe feeding and ruling out risks of microbial infections and spoilage. These seeds served as a baseline, and the processed legume seeds were therefore expected to be approximately the same as their respective raw seeds to confirm stability.

Some processed samples showed slightly higher moisture contents than the raw seeds. For instance, S + I + D (3.53%) versus raw (3.5%) in *C. arietinum*. However, these values are within acceptable limits as similar moisture ranges (2.8% – 3.6%) were reported by Haris et al. (2024), confirming moisture stability post-processing. Moisture contents of the *A. hypogaea* may also not be a cause for concern with a range of 2.9% – 3.64% versus raw seeds (2.82%). Apart from the moisture content of S + M + D (3.64%), which may highlight the synergistic effect of pre-soaking and steam pressure in enhancing moisture absorption, the rest of the moisture contents were found to be within the range of 2.5% – 3.2% as previously reported (Sanni et al. 2024). In addition, I + D resulted in great mineral loss (1.20%), which supports prior observations that infusion promotes dehydration because of osmotic water movement (Kumar et al. 2023).

Similarly, the results for processed *P. vulgaris* with higher moisture than raw (2.5%) had a range of 2.71% – 3.57%. The high moisture content in the processed *P. vulgaris* seeds may be a cause for concern that would require further investigation. Although the C + D (1.58%) and S + C + D (1.05%) methods resulted in low moisture contents, other studies also reported such trends with different processing methods. For instance, Mustapha et al. (2015) reported moisture contents of 5.08% (raw), 3.02% (toasted) and 3.05% (sundried) in *P. vulgaris* seeds.

#### Protein content

Across all processing methods of *C. arietinum, A. hypogaea* and *P. vulgaris*, the highest protein contents of 164.21 g/100 g, 354.31 g/100 g and 453.76 g/100 g were obtained, respectively. In contrast, soaking and autoclaving (S + A + D) led to the lowest protein contents of 309 g/100 g (*P. vulgaris*); 256 g/100 g (*A. hypogaea*) and 108 g/100 g (*C. arietinum*) because of thermal degradation (Dave et al. 2024; Goyal, Thakur & Yadav 2024; Leitzen et al. 2021). These findings are significantly outside the range reported by Hamadou et al. (2022), who observed protein content in soybean whole flour at mean 38.133 s.d. ± 0.366 g/100 g and in the underutilised Bambara bean cultivar BVB at mean 26.434 s.d. ± 0.366 g/100 g. Despite these variations, which may be because of various factors such as legume type, post-harvest processing and geographic locations where the seeds were grown, these findings emphasise the importance of selecting processing methods that balance heat, moisture, and structural modifications to maximise protein retention.

#### Mineral contents

According to DAFF (n.d.), the recommended Zn level for sheep and cattle feed is 18 mg/kg and 27 mg/kg, respectively, while the maximum Zn level of 675 mg/kg and 450 mg/kg is considered tolerable in sheep and cattle, respectively. In this study, the Zn contents of *C. arietinum, A. hypogaea* and *P. vulgaris* were significantly influenced by different processing methods, and their ranges were 33.3 mg/kg – 48.6 mg/kg, 30.9 mg/kg – 51.4 mg/kg and 25.7 mg/kg – 34.5 mg/kg, respectively. Although these ranges are within the tolerable Zn level of both sheep and cattle feed, they are not within the recommended Zn level of sheep feed. Moreover, those of *A. hypogaea* and *C. arietinum* are not within the recommended Zn level of cattle feed. While the C + D (27.4%) was the only method of *P. vulgaris* with the Zn content that is within the recommended level of cattle feed.

Nevertheless, these results are in line with the previous investigations where the three brands of beans had Zn contents of 21 mg/kg – 37 mg/kg (raw), 4.2 mg/kg – 6.4 mg/kg (soaked), and 3.6 mg/kg – 6.6 mg/kg (cooked) (Ramírez-Ojeda, Moreno-Rojas & Cámara-Martos 2018). It is worth noting that the processing methods, such as dehulling, cooking, and drying, reduce the overall mass or moisture content of the legumes, resulting in a concentration effect where minerals appear more abundant per gram (Abbas & Ahmad 2018). Hence, it is not surprising that the result from this study showed the highest Zn contents of 48.6 mg/kg and 34.5 mg/kg because of the cooking process (C + D: *C. arietinum*) and a combined cooking and soaking (S + C + D: *P. vulgaris*), respectively.

Another study reported Zn content variations with different processing techniques that had significant losses because of leaching. However, combined methods such as microwaving, soaking, autoclaving, and infusion helped to retain Zn by stabilising its structure (Khattak et al. 2021; Yegrem 2021). These treatments enhance mineral extractability as they break down seed matrices and reduce antinutritional factors such as tannins, which normally bind minerals (Samtiya, Aluko & Dhewa 2020). Thus, Zn levels may appear higher post-processing despite no mineral addition. Minor variations can also result from natural differences and analytical sensitivity (Ram et al. 2020). Therefore, the high Zn levels observed in *C. arietinum* (I + D; S + D; S + A + D; S + M + D) and *A. hypogaea* (I + D; S + A + D) relative to their raw, may be due to Zn structure stabilisation.

Nonetheless, the Zn content of the raw *A. hypogaea* (41.7 mg/kg) was high when compared to the previous reports. For instance, Zn contents ranging from 17.27 to 31.78 mg/kg were obtained from 14 peanut varieties that were previously investigated (Aşık & Aşık 2023). Another study that conducted a trial of 2000 pigs to analyse the effects of different Zn additives ranging from 10 mg/kg to 100 mg/kg on growth performance, antioxidant performance, immune function and faecal heavy excretion found that Zn supplement of 50 mg/kg had a better average daily gain (ADG), average daily feed intake (ADFI) and feed conversion ratio (FCR) (Ding et al. 2021). Hence, in this study, the *C. arietinum* (C + D [48.6 mg/kg]) and *A. hypogaea* (I + D [51.4 mg/kg]) could possibly give better ADG, ADFI and FCR as their contents are slightly similar to the previous study. Otherwise, all processed and raw seeds of *P. vulgaris* may not have the same results because the highest Zn content (S + C + D) was 34.5 mg/kg, which is relatively low.

According to DAFF (n.d.), the recommended Cu level for sheep and cattle feed is 6.3 mg/kg and 9.0 mg/kg, respectively. While the maximum tolerable Cu levels of 22.5 mg/kg and 90 mg/kg are considered for sheep and cattle, respectively. The different processing methods had an influence on the Cu contents of *C. arietinum, A. hypogaea*, and *P. vulgaris*, which showed ranges of 6.50 mg/kg – 8.12 mg/kg, 5.93 mg/kg – 8.24 mg/kg and 7.83 mg/kg – 9.50 mg/kg, respectively. Even though the Cu contents of the S + M + D (*C. arietinum:* 6.5 mg/kg and *P. vulgaris:* mg/kg) were slightly higher than the recommended Cu level for sheep feed, they were within tolerable levels (sheep and cattle feed). In addition, the S + C + D (6.06 mg/kg) and S + A + D (5.93 mg/kg) of *A. hypogaea* were not within the recommended Cu level of sheep feed, but within the tolerable levels (sheep and cattle).

Conversely, the previous study reported lower Cu contents from raw (5.5 mg/kg – 9.9 mg/kg), soaked (2.5 mg/kg – 4.8 mg/kg) and cooked beans (0.57 mg/kg – 2.6 mg/kg) of three different brands (Ramírez-Ojeda et al. 2018). Another study that analysed the effects of different Cu contents in the diets of the piglets and growing pigs reported the Cu contents of 4 mg/kg – 5 mg/kg that ensure healthy growth and reduction of environmental heavy metal residues (Ding et al. 2021). Although the results from this study were above 5 mg/kg, the processed legume seeds may not benefit the piglets and growing pigs. However, they may play a significant role in the antioxidant status and immune function of weaned piglets. Furthermore, Nazir et al. (2022) reported the Cu content of 2.02 mg/kg – 28.02 mg/kg because of micronutrient profiling of 96 genotypes of *P. vulgaris*. These contents were within the recommended and tolerable Cu levels of sheep and cattle feed and within the range obtained in this study.

The recommended Fe levels for sheep and cattle feed are 27 mg/kg and 450 mg/kg, with maximum tolerable levels of 45 mg/kg and 900 mg/kg, respectively (DAFF n.d.). The Fe contents from the study showed ranges of 59 mg/kg – 101 mg/kg (*C. arietinum*), 26 mg/kg – 55.3 mg/kg (*A. hypogaea*) and 65.3 mg/kg – 86.2 mg/kg. Apart from the three processing methods (C + D [28.9 mg/kg]; S + A + D [28.5 mg/kg]; S + I + D [26.0 mg/kg]) of *A. hypogaea*, the Fe contents of all the methods were not within the recommended levels for sheep and cattle feed. Even though all the processing methods were within the tolerable Fe level for cattle feed, all processed *C. arietinum, P. vulgaris* and *A. hypogaea* (I + D [48.3 mg/kg]; S + M + D [55.3 mg/kg]) were above the level for sheep feed. Even so, the previous study reported lower values of 42 mg/kg – 49 mg/kg (raw), 19 mg/kg – 24 mg/kg (soaked), and 3.9 mg/kg – 16 mg/kg (cooked beans) (Ramírez-Ojeda et al. 2018) to show that the methods in this study were able to retain better Fe contents. Similarly, Huertas et al. (2022) reported Fe levels of 50 mg/kg – 90 mg/kg in six *P. vulgaris* genotypes, still below the recommended level for cattle feed.

The recommended Mg levels for sheep and cattle feed are 0.9 mg/kg – 1.8 mg/kg and 1.0 mg/kg – 2.0 mg/kg, respectively (DAFF n.d.). Whereas the maximum Mg tolerable levels of 4.5 mg/kg and 3.6 mg/kg are considered in sheep and cattle feed, respectively. In this study, the different processing methods affected the Mg contents of *C. arietinum, A. hypogaea*, and *P. vulgaris*, which showed ranges of 0.091 mg/kg – 0.143 mg/kg, 0.174 mg/kg – 0.218 mg/kg and 0.095 mg/kg – 0.111 mg/kg, respectively. The results from the study showed that all the processing methods across the three legume seeds resulted in Mg contents that were below the recommended levels for sheep and cattle feed. However, *A. hypogaea* demonstrated relatively higher Mg values across all processing methods as seen in C + D (0.215 mg/kg), I + D (0.218 mg/kg), S + D (0.200 mg/kg), S + C + D (0.201 mg/kg) and S + A + D (0.208 mg/kg). These values align with those reported by Aşık and Aşık (2023), where Mg level ranged between 0.238 and 0.258 mg/kg in peanut varieties, reinforcing its suitability for inclusion in livestock feed.

### Selection of the best method

Because all the selected methods that were analysed for high nutrient retention were effective in tannin reduction, the results showed that S + M + D reduced the tannins and retained the highest nutrients (62%) in all the legume seeds. To be selected as the best method, it must satisfy three requirements, namely, (1) effective in reducing the tannins; (2) retain high nutrients; (3) cost-effective, especially for rural farming. S + M + D satisfied the first two requirements and not the third requirement, which is of utmost importance for rural farmers in the sub-Saharan African countries with underprivileged infrastructural settings. Therefore, it was not considered the best method. Henceforth, two succeeding methods, S + D (53%) and S + C + D (53%), with tallied nutrient retentions were considered. But then, the processed legume seeds must contain a high amount of protein to be used as protein sources in animal feed and/or feed formulation. Hence, S + D was considered the best method for tannin reduction.

### Strengths and limitations

Strengths of this study include detailed nutrient analysis and rigorous statistical validation, making the results highly applicable to livestock feed formulation. In terms of limitations, the study focused on only three legume seeds, and it was conducted at a laboratory scale. Based on the funding for the study, the proximate and mineral analysis was limited to a few components.

### Implications or recommendations

Processing legumes is essential to reduce tannins and to improve the nutritional quality of the legume seeds. In rural farming, where infrastructure is a major setback for livestock feed, cost-effective methods such as S + D and S + C + D may be considered, as these succeeded the S + M + D in terms of nutrient retention. At an industrial level, the study encourages the application of infusions (I + D; S + I + D) and microwave methods and recommends future research on mineral bioavailability and large-scale validation.

## Conclusion

The processing methods that were effective in the study were selective in reducing the tannins while preserving nutrient-dense legume seeds. Based on the criteria for selection of the best method, soaking plus dehulling was selected as it satisfied all the requirements. Despite its low nutrient retention relative to the soaking plus microwave (62%) method and the fact that it tallied with soaking plus cooking (53%), its application may not compromise feed quality because there are no chemicals or reagents used to boost the processing method that may hamper animal health. Moreover, it showed potential to retain a high amount of proteins in all the legume seeds. Therefore, it can be used to process other legume seeds (not explored in the study) for incorporation in animal feed and/or formulation as safe (from risks associated with high tannins), sustainable and nutritionally rich protein sources.
